# Transcriptomic profiling of tantalum metal implant osseointegration in osteopenic patients

**DOI:** 10.1038/s41405-018-0004-6

**Published:** 2018-11-23

**Authors:** E. K. Hefni, S. Bencharit, S. J. Kim, K. M. Byrd, T. Moreli, F. H. Nociti, S. Offenbacher, S. P. Barros

**Affiliations:** 1 Department of Periodontology, School of Dentistry, University of North Carolina, Chapel Hill, NC USA; 2 Department of General Practice, School of Dentistry, Virginia Commonwealth University, Richmond, VA USA; 3 Department of Periodontology, School of Dentistry, State University of Campinas, Campinas, Brazil

## Abstract

**Objectives:**

The long-term success of dental implants is established by literature. Although clinically well defined, the complex genetic pathways underlying osseointegration have not yet been fully elucidated. Furthermore, patients with osteopenia/osteoporosis are considered to present as higher risk for implant failure. Porous tantalum trabecular metal (PTTM), an open-cell porous biomaterial, is suggested to present enhanced biocompatibility and osteoconductivity. The goal of this study was to evaluate the expression patterns of a panel of genes closely associated with osteogenesis and wound healing in osteopenic patients receiving either traditional titanium (Ti) or PTTM cylinders to assess the pathway of genes activation in the early phases of osseointegration.

**Material and methods:**

Implant cylinders made of Ti and PTTM were placed in osteopenic volunteers. At 2- and 4 weeks of healing, one Ti and one PTTM cylinder were removed from each subject for RT-PCR analysis using osteogenesis PCR array.

**Results:**

Compared to Ti, PTTM-associated bone displayed upregulation of bone matrix proteins, BMP/TGF tisuperfamily, soluble ligand and integrin receptors, growth factors, and collagen genes at one or both time points. Histologically, PTTM implants displayed more robust osteogenesis deposition and maturity when compared to Ti implants from the same patient.

**Conclusions:**

Our results indicate that PTTM properties could induce an earlier activation of genes associated with osteogenesis in osteopenic patients suggesting that PTTM implants may attenuate the relative risk of placing dental implants in this population.

## Introduction

The use of dental implants for the treatment of missing teeth is considered a safe, reliable, and effective alternative to conventional prostheses. Despite the well-documented promise of predictability, however, implant failures still occur.^1–4^ In the clinical environment, the long-term success of dental implants is predicated on the ability to successfully achieve and maintain osseointegration.^5–7^ This phenomenon is both a functional and structural relationship between bone and the outermost surface of a load-bearing implant. Successful dental implant osseointegration has been well-defined clinically and is measured by a lack of increasing relative mobility between the implant and the surrounding trabecular bone after implant placement.^8,9^

Despite some early studies that investigate the complex pathways underlying the phenomenon of osseointegration, the genetic cascade of this process in vivo has yet to be fully elucidated in both healthy human subjects and those presenting with relative risk factors for implant failure.^10–16^ This includes patients presenting with a history of radiation or chemotherapy, smoking, poorly controlled diabetes, and bone metabolic disease such as osteopenia/osteoporosis.^17–21^ A recent meta-analysis of implant failure observed the clear risk for patients with a history of smoking or radiotherapy; however, the report suggested that the risk for patients with diabetes or osteopenia/osteoporosis required additional study.^22^ While several studies have demonstrated that the successful osseointegration of titanium dental implants can be achieved in diabetic individuals with well-controlled glycemic levels,^23,24^ others have reported that the healing process was negatively associated in patients diagnosed with diabetes.^17,25–27^

Chronic bone metabolic disorders such as osteopenia and osteoporosis are highly prevalent in older patients and are expected to increase in prevalence as patients have longer life expectancy.^28–31^ Osteopenia, previously referred to as low bone density or low bone mass, is defined by lower bone mineral density (BMD) T scores (grams of mineral per area or volume) between −2.5 and −1.0. Using this classification, nearly 50% of all women over 50 years old are osteopenic compared with 10% of the population suffering from more severe category of osteoporosis.^30,32–34^ Osteoporosis is characterized by altered trabecular bone strength, reduced capacity for bone regeneration and has been reported to present a risk for implant failure.^35,36^ Epidemiologically, women with osteopenia/osteoporosis are at significantly higher lifetime risk of partial edentulism when compared to women with normal BMD.^37,38^ In vivo modeling of osteoporosis in rats displayed less bone-implant contact and lower BMD; however, the current literature on the success of dental implants in osteopenia/osteoporotic patients is often contradictory.^39–44^ Thus, osteopenia/osteoporosis is not considered an absolute contraindication for implant placement;^28,45,46^ however, any concurrent medical histories including smoking, current, and recent radiotherapy, or the use of antiresorptive drugs present an additional risk of implant failure.^22,47,48^

For years, improving osseointegration among healthy and at-risk patients has been a significant goal. This has led to improvements in both surgical protocols and implant design, including changes to the chemistry or topography of the implant surface.^49,50^ Recently, focus has shifted to the application of porous tantalum trabecular metal (PTTM) as a surface and design for titanium implants, both orally and orthopedically.^51–57^ Tantalum metal displays superior biocompatible and its biochemical, and biomechanical properties that encourage osseointegration just as titanium. Unlike the rigid titanium, tantalum metal has a modulus of elasticity comparable to the surrounding trabecular bone. Tantalum metal also presents with improved frictional properties and is characterized by a high resistance to acid corrosion.^51,58,59^ In addition, when tantalum metal is utilized as a dental implant surface enhancement, it is manufactured to mimic the three-dimensional, open-cell structure of trabecular bone.^60,61^ Such porosity allows for its enhanced osteoconductivity and neovascularization, and permits bone to actually anchor onto the outer surface and inside the interconnected pores of PTTM.^62–70^ What remains unknown is whether PTTM implants are able to more robustly induce osseointegration in patients with relative risk factors.

In this study, we selectively examined genetic pathways associated with osseous wound healing using real-time polymerase chain reaction (RT-PCR) and histology to compare the PTTM vs. titanium metal implants in osteopenic patients. Here we show both genetically and histologically that osteopenic patients treated with PTTM implants display enhanced osseous wound healing by early regulation of specific osseoinductive factors that leads to a higher deposition of bone and bone density as compared to titanium implants. Our results suggest that the use of PTTM implants may induce an earlier osseointegration cascade among osteopenic patients that may counter the relative risk of placing dental implants in this population.

## Materials and Methods

### Subjects

The Institutional Review Board (IRB) of the University of North Carolina at Chapel Hill approved this study. Written informed consent was obtained from all study participants prior to treatment. Patient selection criteria are presented in Table 1.

**Table 1:** Inclusion and exclusion criteria

Inclusion criteria:
Subjects must be adult males or females between the age of 18 and 80 years (inclusive).
Subjects must be able and willing to follow study procedures and instructions in English.
Subjects must have read, understood and signed an informed consent form in English.
Subjects must have at least two mandibular implants as their future treatment needs.
Subjects must meet one of the following categories to be considered for enrollment:
Osteoporosis or osteopenia patient: Subjects must be diagnosed with osteoporosis or osteopenia and must be currently under the care of a physician and treatment with oral bisphosphonates. Subjects must have never had intravenous (IV) bisphosphonates. Subjects in this group must be non-diabetic and no history of smoking within the last 2 years.
Exclusion criteria:
Individuals who have a chronic disease with oral manifestations.
Individuals who exhibit gross oral pathology.
The use of either antibiotics or chronic use of NSAIDs within 1 month prior to screening examination.
Individuals that require antibiotic prophylaxis prior to dental treatment.
Chronic treatment (i.e., two weeks or more) with any medication known to affect periodontal status (e.g., phenytoin, calcium antagonists, cyclosporine, Coumadin) within 1 month prior to screening examination.
Systemic conditions, except osteoporosis and osteopenia that are known to affect periodontal status.
Individual with uncontrolled parafunctional habits, such as clenching and bruxing on objects, that could adversely impact implant survival.
Individuals with a history of intravenous bisphosphonates Individuals with active infectious diseases such as hepatitis, HIV or tuberculosis.
Individuals with a current tobacco use history.
Individuals who are pregnant, breastfeeding or planning to become pregnant within 3 months.

### Surgical procedures and bone biopsy collection

Radiographs demonstrated that all subjects had partially edentulous mandibular ridge areas in adequate dimensions to accommodate 2 test and 2 control cylinders (3 × 5 mm each) placed on each side of the mandible. Ti and PTTM study cylinders were placed level with the crestal bone and covered with a collagen membrane (BioMend, Zimmer Biomet Dental, Palm Beach Gardens, FL). After placement, a 5.0 mm-diameter tissue punch and a 4.5 mm-diameter trephine drill were used to explant 1 Ti and 1 PTTM test cylinder from each side of the mandible after 2 and 4 weeks of healing.

After explantation, each cylinder was placed separately into a microfuge tube containing RNA stabilization solution (Ambion RNAlater^**®**^ Tissue Collection, Thermofisher Scientific, Waltham, MA) and temporarily stored at 4 °C overnight. The next morning, the solution was decanted and the samples were flash-frozen in liquid nitrogen and stored at −80 °C until RNA isolation.

### RNA isolation

Bone tissue surrounding each study cylinder was collected and homogenized in liquid nitrogen using sterile mortar and pestle and liquid nitrogen. Total RNA was isolated from the bone biopsy using RNeasy Mini kit (Qiagen, Cat. No. 217084), according to the manufacturer's instructions. Samples were eluted in 30 μL nuclease-free water (Qiagen) and stored at −80 °C. RNA quality and quantity were analyzed using a spectrophotometer (NanoDrop ND-1000, Thermo Scientific, Wilmington, DE) and digital analyzer (Agilent 2100 Bioanalyzer, Agilent Technologies, Inc., Waldbronn, Germany).

### Quantitative Real-time PCR

For each sample, a volume of 300 ng of RNA was used to generate complementary DNA (cDNA) through reverse transcription reactions using a first-strand cDNA synthesis technique (RT^2^ First Strands Kit, Qiagen). Genes of interest related to human osteogenesis were examined using a gene array (RT^2^ Profiler™ PCR Array Human Osteogenesis, PAHS-026Z, Qiagen) and RT-PCR was performed (7500 Sequence Detection system, ABI prism, Applied Biosystems, ThermoFisher Scientific). The human osteogenesis panel included the following functional gene groups: skeletal development, bone mineral metabolism, cell growth and differentiation, extracellular matrix molecules, and transcription factors and regulators. The mRNA expression levels were normalized using multiple housekeeping genes (GAPDH, HPRT1, GUSB), and the fold changes were calculated by means of 2^−∆∆CT^ method on each group.^71^

Fold change values were calculated by comparing the normalized copy number of individual samples with the mean of the control samples.

### Statistical analysis

To compare gene expression in different groups, we used web-based RT2 Profiler PCR Array Data Analysis, (http://pcrdataanalysis.sabiosciences.com/pcr/arrayanalysis.php). This web-based analysis by default employs Student’s *t* test to examine the differences between groups, We applied the false discover rate test to calculate statistical significance set at *p* < 0.05.

### Ingenuity pathway analysis

The canonical pathways, regulator effects and networks function included in IPA (Ingenuity System Inc, USA) were used to interpret the data in the context of biological processes, pathways and networks. Both up- and downregulated identifiers were defined as value parameters for the analysis. After the analysis, generated networks associated with function appear ordered by a score meaning significance.

### Histology

The tissue blocks from the implants were prepared for ground sectioning. The samples were transferred to 0.1M Cacodylate buffer, pH 7.4, for several hours to overnight. Dehydration was started with an ethanol series: 50%, 70%, 95% ethanol in distilled water for 10 min each. They were then transferred into absolute ethanol for two rinses of 20 min each. The samples were infiltrated with a 50:50 mixture of Polybed resin (Polysciences Inc, Warrenton, PA) and absolute ethanol for 6–12 h. They were then embedded with several changes of pure resin into BEEM® capsules and cured overnight at 65 degrees C. The orientation of the samples during embedment was carefully maintained to facilitate cross sectioning of the implants. Cured resin blocks containing the implants were removed from the polyethylene capsules and were sectioned following the long axis of the implants using a diamond band saw fitted in a precision slicing machine (Microslice 2TM; Ultratec, Santa Ana, CA, USA) a thickness of ~50–60 μm. Two central sections were harvested and then hand-polished and thinned using water proof paper. Histological slides were stained with toluidine blue and examined under confocal microscope.

## Results

Eight osteopenic female’s subjects aged between 57 and 76 years were included in this study. A total of 32 experimental cylinders (16 Ti control cylinders and 16 PTTM test cylinders) were placed. Each subject received 2 adjacent test cylinders and 2 adjacent control cylinders on opposite’s sides of the same jaw. At each time point, one Ti and one PTTM test cylinder were retrieved from each subject. Transcript analysis was performed on all 32 (16 test and 16 control) samples using PCR array panels. To understand the potential mechanisms involved in the regulatory effect of the PTTM cylinder on osteopenic patients, 84 genes related to osteogenic differentiation were profiled. Growth factors and genes mediating osteogenesis and related cell growth, proliferation, and differentiation processes were included, and categorized as bone matrix proteins, BMP/TGF superfamily genes, soluble ligand receptors, growth factors, integrin receptors, collagen, cartilage-related genes, metalloproteinase, and transcription factors (Table 2). PTTM cylinders were compared to Titanium cylinders in osteopenic patients at 2-week and 4-week time points, for the fold changes values presented in Table 2, titanium samples were used as control in the statistical analysis, therefore genes up or downregulation indicate how PTMM compares to Ti for each gene and period of evaluation, 2 or 4 weeks. Specific gene regulation was observed for most of the studied genes. An increase in gene expression and number of genes from this panel at 4 weeks was observed as follows:

**Table 2:** Gene regulation PTTM compared to titanium test cylinders (controls) in osteopenic subjects

Gene	Description	2 weeks		4 weeks	
	Bone Matrix Proteins:	Fold change	*p* value	Fold change	*p* value
ALPL	Alkaline phosphatase	1.2958	n/s	↑ 10.267	n/s
BGLAP	Osteocalcin	1.1872	n/s	↑ 3.8334	n/s
BGN	Biglycan	1.2798	n/s	0.687	n/s
	BMP/TGF Superfamily				
BMP2	Bone morphogenetic protein 2	0.8331	n/s	1.2338	n/s
BMP3	Bone morphogenetic protein 3	↓ 0.3306	n/s	1.8761	n/s
BMP4	Bone morphogenetic protein 4	↑2.3184	n/s	1.176	n/s
BMP5	Bone morphogenetic protein 5	1.9941	n/s	0.7928	n/s
BMP6	Bone morphogenetic protein 6	↓ 0.4085	n/s	1.3073	n/s
GDF10	Growth differentiation factor 10	1.1357	n/s	1.4289	n/s
TGFB1	Transforming growth factor, beta 1	1.5316	n/s	0.9806	n/s
TGFB2	Transforming growth factor, beta 2	1.5557	n/s	↑ 6.0062	n/s
TGFB3	Transforming growth factor, beta 3	↑2.2786	n/s	1.0549	n/s
	Receptors				
CALCR	Calcitonin Receptor	↓0.3881	n/s	1.1758	n/s
CD36	CD36 molecule (thrombospondin receptor)	↑ 2.705	n/s	↑ 8.7996	n/s
CDH11*	Cadherin 11, type 2, OB-cadherin (osteoblast)	↑ 2.023*	0.04	1.3055	n/s
EGFR	Epidermal growth factor receptor	1.1334	n/s	↑ 3.9846	n/s
FGFR1	Fibroblast growth factor receptor 1	1.1701	n/s	0.7648	n/s
FGFR2	Fibroblast growth factor receptor 2	↑2.3888	n/s	↑ 4.2764	n/s
FLT1	Fms-related tyrosine kinase 1	1.6261	n/s	↑ 2.0665	n/s
ICAM1	Intercellular adhesion molecule 1	0.5341	n/s	0.7289	n/s
TGFBR1	Transforming growth factor, beta receptor 1	1.5316	n/s	0.872	n/s
TGFBR2	Transforming growth factor, beta receptor II (70/80kDa)	1.5557	n/s	1.6413	n/s
VCAM1	Vascular cell adhesion molecule 1	↑ 2.1988	n/s	0.5218	n/s
VDR	Vitamin D (1,25- dihydroxyvitamin D3) receptor	↓ 0.4113	n/s	↑ 2.4299	n/s
IGF1R	Insulin-like growth factor 1 receptor	1.7216	n/s	1.2822	n/s
PHEX	Phosphate regulating endopeptidase homolog, X-linked	↓ 0.2887	n/s	↑ 12.973	n/s
	Growth factors				
EGF	Epidermal growth factor	↓ 0.1831	n/s	↑ 2.9981	n/s
FGF1	Fibroblast growth factor 1 (acidic)	1.1701	n/s	1.5146	n/s
FGF2	Fibroblast growth factor 2 (basic)	2.3888	n/s	1.0899	n/s
IGF1	Insulin-like growth factor 1 (somatomedin C)	1.0508	n/s	0.8316	n/s
IGF2	Insulin-like growth factor 2 (somatomedin A)	1.8386	n/s	1.3747	n/s
PDGFA	Platelet-derived growth factor alpha	1.4664	n/s	1.1025	n/s
VEGFA	Vascular endothelial growth factor A	1.7326	n/s	1.3852	n/s
VEGFB	Vascular endothelial growth factor B	1.4688	n/s	↑ 3.5104	n/s
	Integrin receptors				
ITGA1	Integrin, alpha 1	↑ 2.7695	n/s	↑ 3.2849	n/s
ITGA2	Integrin, alpha 2	0.9068	n/s	↑ 3.1185	n/s
ITGA3	Integrin, alpha 3	0.8153	n/s	1.3617	n/s
ITGAM	Integrin, alpha	0.8984	n/s	0.698	n/s
ITGB1*	Integrin, beta 1	↑ 2.6248*	0.03	1.398	n/s
	Collagens				
COL10A1	Collagen, type X, alpha 1	0.9751	n/s	1.1104	n/s
COL14A1	Collagen, type XIV, alpha 1	0.6952	n/s	0.9267	n/s
COL15A1	Collagen, type XV, alpha 1	↑ 3.2336	n/s	0.6764	n/s
COL1A1	Collagen, type I, alpha 1	↑ 2.1891	n/s	1.042	n/s
COL1A2	Collagen, type I, alpha 2	↑ 4.9625	n/s	1.928	n/s
COL2A1	Collagen, type II, alpha 1	0.6156	n/s	↑ 2.3359	n/s
COL3A1*	Collagen, type III, alpha 1	↑ 5.7326 *	**0.02**	0.6582	n/s
COL5A1	Collagen, type V, alpha 1	1.9995	n/s	1.7454	n/s
	Cartilage-related genes				
COMP	Cartilage oligomeric matrix protein	↑ 4.3585	n/s	0.6924	n/s
SOX9	SRY (sex determining region Y)-box 9	↓ 0.28	n/s	↑ 2.3788	n/s
	Metalloproteinases				
BMP1	Bone morphogenetic protein 1	0.8346	n/s	1.2873	n/s
MMP10	Matrix metallopeptidase 10 (stromelysin 2)	↓ 0.0461	n/s	↑ 2.9981	0.2
MMP2	Matrix metallopeptidase 2 (gelatinase)	1.0813	n/s	1.1501	n/s
MMP8	Matrix metallopeptidase 8 (neutrophil collagenase)	↓ 0.1815	n/s	1.5924	n/s
MMP9	Matrix metallopeptidase 9 (gelatinase B)	0.9976	n/s	0.6249	n/s
	Transcription Factors				
NFKB1	Nuclear factor of kappa light polypeptide gene enhancer in B-cells 1	↓ 0.4659	n/s	-1.4106	n/s
RUNX2	Runt-related transcription factor 2	1.1996	n/s	1.0036	n/s
SMAD1	SMAD family member 1	1.2415	n/s	↑ 2.3788	n/s
SMAD2	SMAD family member 2	1.3418	n/s	1.2641	n/s
SMAD3	SMAD family member 3	↓ 0.3437	n/s	1.1738	n/s
SMAD4	SMAD family member 4	0.6867	n/s	1.8916	n/s
TWIST1	Twist homolog 1 (Drosophila)	1.0944	n/s	↑ 7.0499	n/s
	Other genes				
CTSK	Cathepsin K	1.9511	n/s	0.5538	n/s
**FN1**	Fibronectin 1	↑ 2.8355*	**0.02**	1.0589	n/s
SERPINH1	Serpin peptidase inhibitor, clade H (heat shock protein 47), member 1, (collagen binding protein 1)	1.7138	n/s	0.7208	n/s

Bold values indicate statistical significance for fold changes

### Bone matrix proteins

**Fig. 1:**
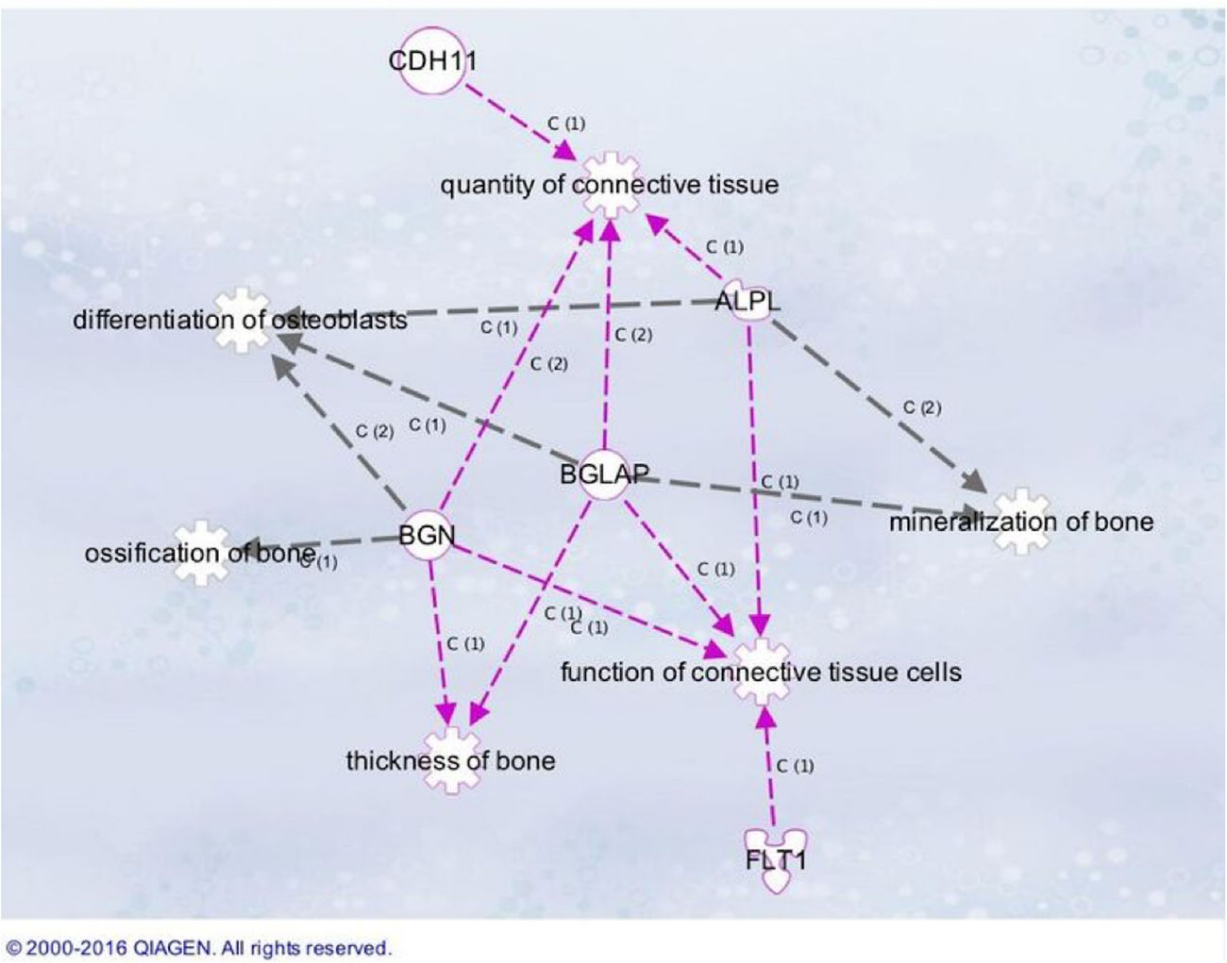
Ingenuity pathway analysis indicating functional properties of selected transcripts differentially regulated during healing when PTTM related bone samples were compared to Ti's. Each interaction is supported by literature references in the IPA Knowledge Base

The levels of ALPL and BGLAP mRNA were upregulated at 4 weeks. BGN mRNA was also evaluated in this study and levels were unchanged up to week 4. Figure 1 indicates the pathway associated with gene function.

### BMP/TGF superfamily

At 2-week upregulation for BMP4 and TGFB3 mRNA expression was observed, but not at the later time point. Downregulation of BMP3 and BMP6 was observed at 2 weeks but not at the 4-week time point. The TGFB2 levels were also increased (6-fold increase) at 4 weeks but not at 2 weeks.

### Soluble ligand receptors

The expression level of 14 mRNAs encoding receptors associated with different functions during cell differentiation is shown in Table 2. Calcitonin receptor mRNA expression levels decreased at 2 weeks then increased at 4 weeks. CD36 and FGFR2 mRNA presented an increased expression at 2 and 4 weeks. CDH11 and VCAM1 mRNA expression levels were upregulated at 2 weeks but not at 4 weeks, while FLT1 mRNA expression levels were upregulated at 4 weeks but not at 2 weeks. Upregulation of EGFR, VDR, and PHEX were observed at the later timer point.

### Growth factors

Compared to the Ti group, the PTTM group exhibited higher expressions of the following growth factors: FGF2 at 2 weeks, and EGF at 4 weeks. VEGFB mRNA expression was 3.5-fold higher than the Ti group at 4 weeks.

### Integrin receptors

ITGA1 mRNA levels were upregulated at 2 and 4 weeks, with a 2.8- and 3.3-fold increase, respectively. ITGA2 mRNA levels were upregulated at 4 weeks. At 2 weeks, statistically significant ITGB mRNA levels were upregulated in the PTTM implant group compared to the Ti group in osteopenic patients.

### Collagen genes

COL15A1, COL1A1, COL1A2, and COL3A1 mRNA levels were upregulated at 2 weeks but the COL3A1 was statistically significant upregulated on PTTE implant compared to Ti in osteopenic patient. An increased mRNA expression levels for COL2A1 at 4 weeks was also found.

Other genes evaluated in this study were Cathepsin K (CTSK), SERPINH1, and Fibronectin 1 (FN1). FN1 expression levels, which were increased 2.8-fold at week 2, at a statistical significant level (*p* = 0.02).

**Fig. 2:**
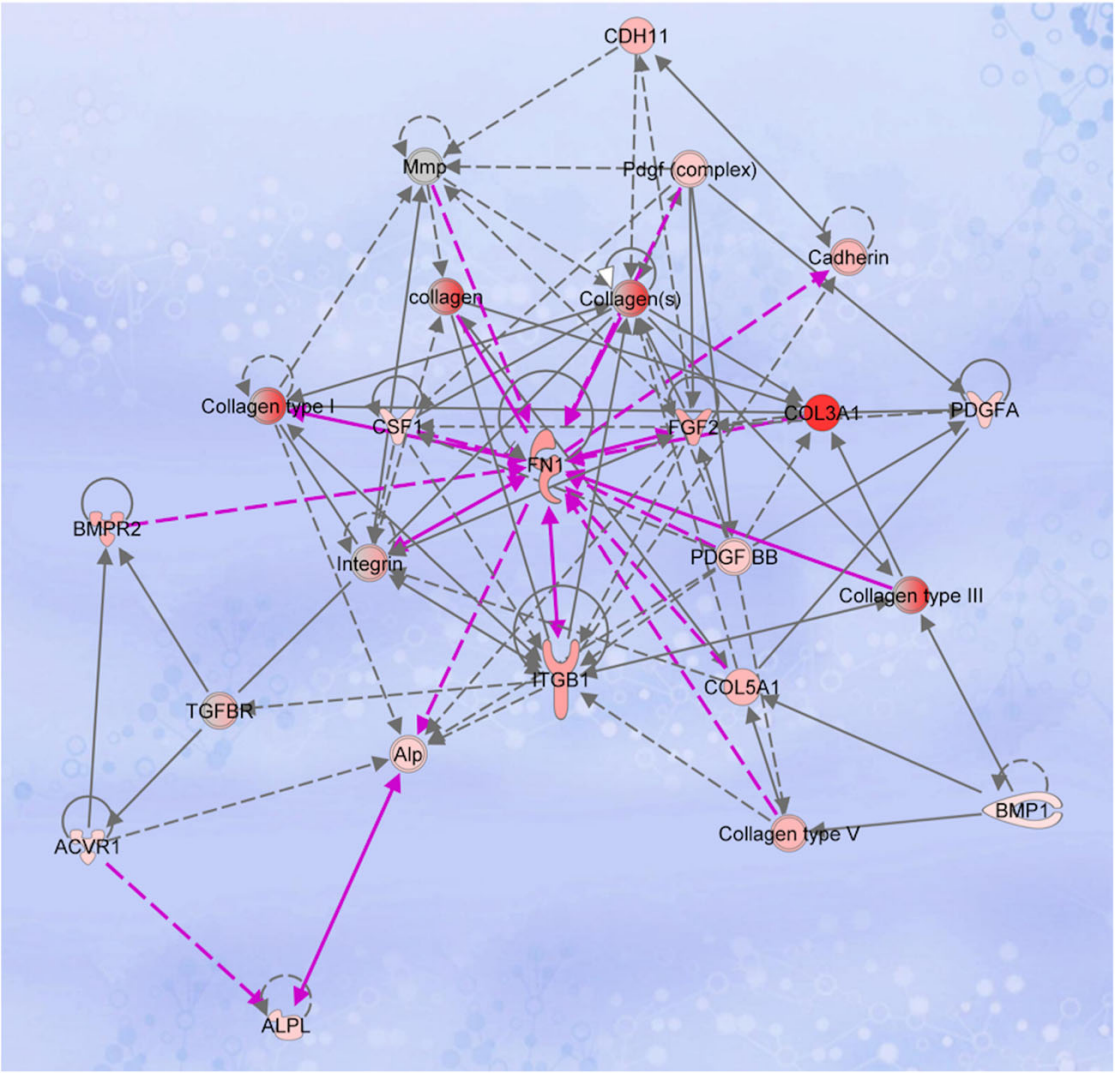
Ingenuity Pathway Analysis (IPA) showing network relevant to extra cellular matrix formation. Transcripts highlighted in red were upregulated in the comparison of PTTM relative to Ti related bone samples at week 2 of healing. Fibronectin (FN1) interactions with collagens and other transcripts associated with cell adhesion, growth and differentiation are shown. Each interaction is supported by literature references in the IPA Knowledge Base. Solid lines represent direct interactions and dashed lines represent indirect interactions

**Fig. 3:**
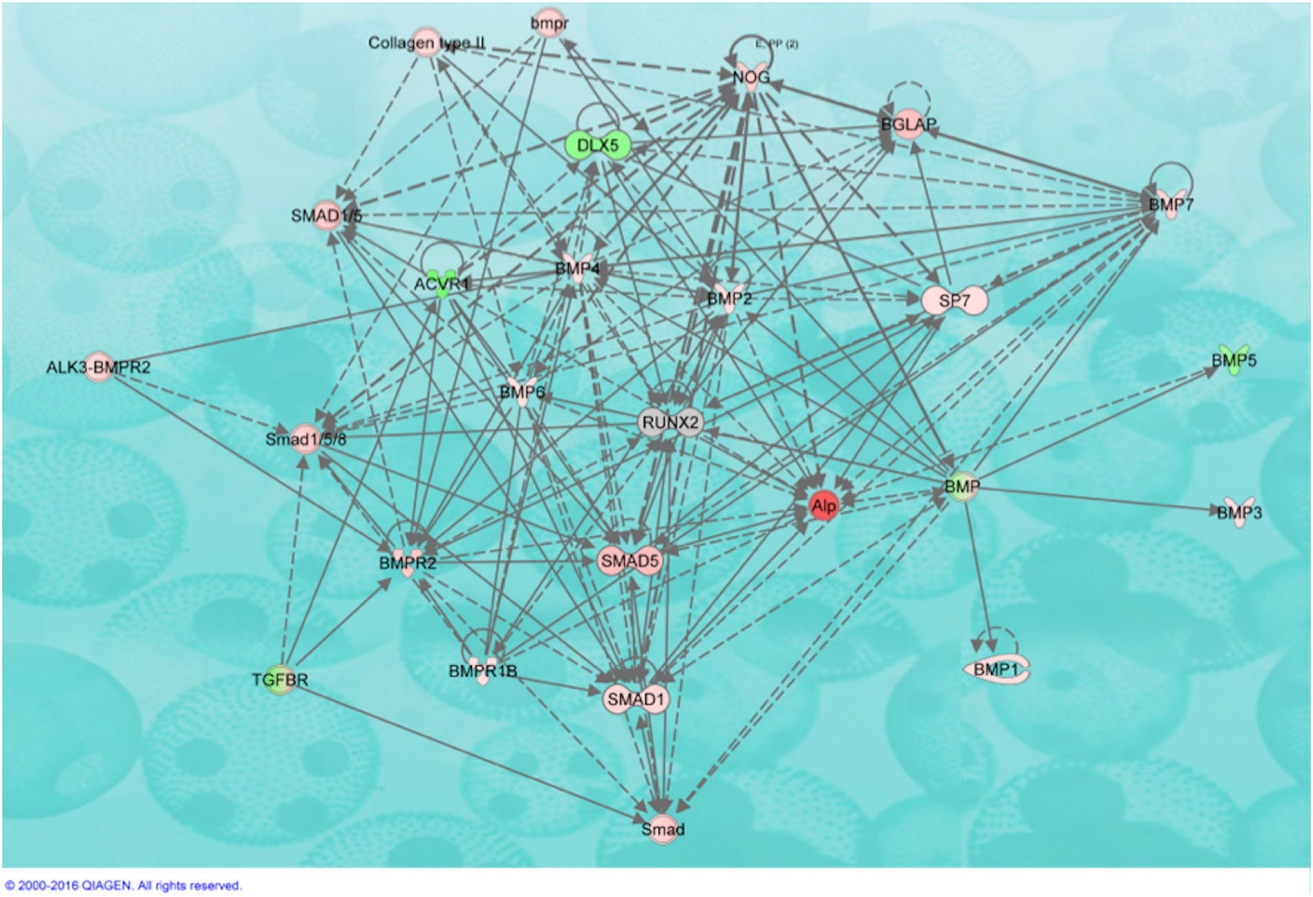
Molecules in network for cellular and tissue development at 4 weeks of wound healing comparing tantalum to titanium in osteopenic patients. Ingenuity Pathway Analysis (IPA) relevant to cellular growth and proliferation. Transcripts that were upregulated in the comparison between PTTM relative to Ti associated bone samples are shown in red and downregulated molecules are displayed in green. Each interaction is supported by literature references in the IPA Knowledge Base. Solid lines represent direct interactions and dashed lines represent indirect interactions

Networks with genes in play are shown in Fig. 2 corresponding to week 2 and Fig. 3 corresponding to week 4 in osteopenic patients from tantalum group in comparison to titanium.

## Discussion

Currently, titanium is the most commonly used material for dental implants because of its superior biocompatible, biochemical, and biomechanical properties, and documented long-term clinical success.^72,73^ However, during recent decades continuously increasing numbers of biomedical implants have been introduced for use in the human body, and the interdisciplinary field of biocompatible implant surfaces from the viewpoint of materials science, biochemistry, and cell biology has been explored in search for materials and surfaces with optimum modulus of elasticity, shear strength, and frictional properties. And although systemic diseases, such as diabetes, endocrine pathologies, or controlled metabolic disorders do not seem to be a total or partial contraindication to the placement of dental implants, such factors have prompted continuing search for improved biomaterials that may also promote long-term dental implant stability through more efficient osseointegration.^31,74^

Osteoporosis, as a metabolic disease which modifies the bone mass and density, is the most frequent bone disorder, which affects sponge bone mainly and is more common in postmenopausal women. It has been considered for a long time that this osteoporosis could complicate the initial stability of dental implants due to potential loss in the bone mass. Although reported short term implant survival rates have reported to average 93.8%, with a trend but no statistical significant association between osteoporosis and implants failures.^75^

We conducted this investigation involving different implant surfaces in a group of subjects with such bone disorder aiming to identify potential differences regarding time of pro-osteogenic cell signaling pathways activation and initial healing.

We should also point out that the osteopenic individuals in our study were under treatment with oral bisphosphonates, a class of drugs indicated in the prevention and treatment of illnesses associated to bony resorption. Bisphosphonates have shown to be highly effective inhibitors of bone resorption that selectively affect osteoclasts in vivo.^76^ In vitro study with gingival fibroblasts exposed to bisphosphonate have indicated the upregulation of VEGFA, and BMP2 genes.^77^

Also we should note that in our analysis the comparison between different implants/surfaces was measured in individuals who received both implants types and all of them were under oral bisphosphonate treatment. However, we recognize that a limitation of our study is not to account for the specific bisphosphonates, used by each study participant.

Our data presented the transcriptional analysis of osteopenic subjects comparing gene expression profiles associated with healing and osseointegration at 2 and 4 weeks for experimental cylinders made of PTTM (test) and Ti (control). Results for the PTTM implant suggest a general trend of upregulation of the genes in the osteogenic pathways and a more discrete expression of genes regulating bone resorption. As indicated in Fig. 1 that highlights genes related to osteoblast differentiation and mineralization, a 10-fold upregulation of the Alkaline phosphatase gene (ALP) was observed for PTTM compared to Ti cylinders in osteopenic patients. Evidence that tissue-nonspecific alkaline phosphatase plays an important role in normal biomineralization has been documented in the literature.^78–80^ ALP is among the first functional genes expressed in the process of calcification. It is therefore likely that at least one of its roles in the mineralization process at an early phase. A clue to the role of ALP in calcification came from studies in subjects displaying hypophosphatasia, whose disease resulted from missense mutations in the gene coding for tissue-nonspecific alkaline phosphatase, which led to decreased or absent ALP activity.^81^ Another gene implicated in the regulation of early osteogenesis is Bone gamma-carboxyglutamate Protein/Osteocalcin (BGLAP), which is a key component of the early extracellular matrix necessary bone formation.^82^ Osteocalcin is a bone-specific protein that comprisesabout 15% of the noncollagenous protein component of bone^83^ is a highly conserved bone-specific protein that is synthesized by osteoblasts and the Osteocalcin (BLGAP) 3.8-fold upregulation at 4 weeks in PTTM-associated bone samples, indicates the relative increased expression of *Bglap*, which is normally detected in mature osteoblasts and also osteocytes in ossifying centers.^84^ Osteocalcin detection early in the process of mineralization suggest a fundamental role for osteocalcin in attainment and maintenance of the bone mineral matrix, as well as in bone remodeling.^85^ It is highly conserved across species and has numerous regulatory elements among them the vitamin D receptor, which positively regulates the transcription by binding to the osteocalcin promoter.^86^ In our results, Vitamin D receptor was upregulated (2.43 fold) at week 4 in bone samples collected from PTTM test cylinders.

Figures 1 and 2 also illustrate ALP, BGN (biglycan), and BMP4 networks linked to bone maturation. BGN modulates osteoblast differentiation by regulating bone morphogenetic protein-4 (BMP-4), Biglycan signaling promotes osteoblast differentiation and it has been shown that BGN knockout mice have an age-dependent osteoporosis-like phenotype, including a reduced growth rate and lower bone mass due to decreased bone formation.^87^ It has also been shown in mice that biglycan deficiency caused less BMP-4 binding, which subsequently reduced the sensitivity of osteoblasts to BMP-4 stimulation, ultimately leading to a defect in the differentiation of osteoblasts.^88^ BMP4 is recognized as one of the most potent inducers of bone formation through its stimulation of osteoblast differentiation.^89^ Regarding the TGF/BMP superfamily, our results showed 2.3 fold increase in BMP4 expression for the PTTM in comparison to the Ti associated bone samples at 2 weeks. TGFB2 and TGFB3 are other factors involved in osteoblast differentiation and bone formation,^90^ and were also upregulated in the PTTM-associated bone. Although an increase in TGF/BMP superfamily genes was observed, there were no significant changes in SMAD transcription factor genes. We also found that levels of FLT1 in the PTTM-related samples were increased at 4 weeks. Otomo et al. ^81^ demonstrated that Flt-1 tyrosine kinase deficiency leads to decreased trabecular bone volume with reduced osteogenic potential by demonstrating that disruption of the FLT1 tyrosine kinase domain gene promoted significant reduction in the mineralizing surface, mineral apposition rate, and bone formation rate in the trabecular bone in animal model.

Table 3 summarizes the functional properties of the differentially expressed target genes, those significantly upregulated genes are also shown in play through the network generated by Ingenuity Pathway Analysis as seen in Fig. 2 for PTTM-related samples. Our data also indicates that PTTM-related bone samples exhibited a statistically significant increase in CHD11 levels at 2 weeks. Previous studies have established that osteoblasts express mostly N-cadherin (cadherin-2, CDH2) and cadherin-11 (CDH11), known to be upregulated during osteoblast differentiation. The expression of this particular cadherin with upregulation during differentiation, suggests a specific function in bone development and maintenance of bone mass.^91^

**Table 3:** Functional properties of statistically differentially expressed target genes

Gene Symbol	Gene name	Function
CDH11*	Cadherin 11	Calcium-dependent cell adhesion proteins
ITGB1*	Integrin Subunit Beta 1	Fibronectin receptor, involved in osteoblast compaction through the fibronectin fibrillogenesis cell-mediated matrix assembly process and the formation of mineralized bone
COL3A1*	Collagen Type III Alpha 1 Chain	Bone-specific collagen known to be expressed in immature bone
FN1	Fibronectin 1	Cell adhesion and migration during wound healing

The healing process of alveolar bone after implantation necessitates adequate production and interpretation of network of local growth factors. Growth factors that are involved in the commitment of mesenchymal stem cells (MSCs) to osteoblastic lineage, osseoinduction, and vascularization, play critical roles in determining the success of the bone healing, and play a role on osseointegration.^92^

PTTM-related samples also showed upregulation of genes associated with angiogenesis (FGF2, VEGFB, TNF, EGF, IGF1, and ITGB1) (Fig. 2). The importance of angiogenesis in bone healing and regeneration is well recognized and represents another potential target mechanism for modulating the osseointegration process.^93^

The effects of extracellular matrix proteins on the growth of bone cells are mediated mainly via integrin receptors. Integrins form a part of the focal adhesion process that is primarily responsible for cell attachment and spreading by physically linking integrins to actin cytoskeleton.^94^ PTTM-related samples showed an increase and upregulation of mRNA expression of different integrin receptor ITGA1 and ITGA2 and ITGFGB1, suggesting the promotion of early healing and tissue adhesion associated with PTTM.

Most of the collagens were upregulated either at 2 or 4 weeks. COL1A1 and COL3A1 are both localized in bone tissue, and these genes are upregulated in the early stages of osteoblast differentiation.^95^ These findings suggest that the critical events associated with bone formation during the process of osseointegration are influenced by the surface of the implant, and in particular by the cell–implant interface. Within the limitations of the present study, PTTM exhibited a more robust response towards early bone formation and mineralization, which may potentially enhance early osseointegration.
